# Social signals and algorithmic trading of Bitcoin

**DOI:** 10.1098/rsos.150288

**Published:** 2015-09-23

**Authors:** David Garcia, Frank Schweitzer

**Affiliations:** Chair of Systems Design, ETH Zurich, Weinbergstrasse 56/58, Zurich 8092, Switzerland

**Keywords:** Bitcoin, computational social science, algorithmic trading, polarization, sentiment, prediction

## Abstract

The availability of data on digital traces is growing to unprecedented sizes, but inferring actionable knowledge from large-scale data is far from being trivial. This is especially important for computational finance, where digital traces of human behaviour offer a great potential to drive trading strategies. We contribute to this by providing a consistent approach that integrates various datasources in the design of algorithmic traders. This allows us to derive insights into the principles behind the profitability of our trading strategies. We illustrate our approach through the analysis of Bitcoin, a cryptocurrency known for its large price fluctuations. In our analysis, we include economic signals of volume and price of exchange for USD, adoption of the Bitcoin technology and transaction volume of Bitcoin. We add social signals related to information search, word of mouth volume, emotional valence and opinion polarization as expressed in tweets related to Bitcoin for more than 3 years. Our analysis reveals that increases in opinion polarization and exchange volume precede rising Bitcoin prices, and that emotional valence precedes opinion polarization and rising exchange volumes. We apply these insights to design algorithmic trading strategies for Bitcoin, reaching very high profits in less than a year. We verify this high profitability with robust statistical methods that take into account risk and trading costs, confirming the long-standing hypothesis that trading-based social media sentiment has the potential to yield positive returns on investment.

## 1. Introduction

Our online society generates data on the digital traces of human behaviour at unprecedented scales and resolutions. This produces a *data deluge*, in which researchers are confronted with a vast amount of observational data that is not the product of carefully designed experiments [1]. One of the main challenges of the scientific community is to develop methods to extract meaningful knowledge from that data beyond mere descriptive analyses [2]. This is particularly important in financial trading: data can be available to all financial agents, but it is the analysis and its applications which makes a difference. Within computational finance, the field of algorithmic trading [3] deals with the implementation and evaluation of automatic trading strategies, which are often kept in private companies and away from publicly accessible research. The most common kind of algorithmic trading is based on the principles of *technical analysis* [4], using the time series of prices to formulate predictions about returns. Technical analysis is often insufficient to derive satisfactory returns [5], motivating the inclusion of large-scale social signals and the evaluation through data-driven simulations on historical data, called *backtesting* [6,7]. In this article, we present a set of methods to derive stylized facts from the analysis of multidimensional economic and social signals, and to apply that knowledge in the design and evaluation of algorithmic trading strategies. We illustrate an application of our approach to algorithmic trading of the Bitcoin cryptocurrency, using a wide variety of digital traces about economic and social aspects of the Bitcoin ecosystem.

Bitcoin (BTC) is a digital currency designed to operate in a distributed system without any central authority, based on a cryptographic protocol that does not require a trusted third party [8]. Introduced in a 2008 paper written under the pseudonym of Satoshi Nakamoto [9], Bitcoin serves as a technology to transfer money quickly for negligible fees [10]. One of the first markets to adopt Bitcoin was the *Silk Road*, a website where illegal commerce became possible thanks to the relative anonymity of Bitcoin [11], in line with the evidence in search trends that relates Bitcoin usage to computer expertise and illegal activities [12]. Since then, the use of Bitcoin has widely expanded beyond criminal activities: at the time of writing, Bitcoin is accepted by many legal merchants and charities [13], including large businesses like Dell [14]. Bitcoin-accepting businesses, exchange markets and wallet services compose the *Bitcoin ecosystem* [8], where different kinds of agents interact, trade and communicate through digital channels. The increasing adoption of Bitcoin and its online nature allow us to simultaneously monitor its social and economic aspects. Every purchase of goods or services in Bitcoin leaves a trace in a public ledger called the *Block Chain*, creating a publicly accessible economic network [15]. Bitcoin's delocalized technology aligns with the online interaction of its users through social networks and forums, motivating its adoption by new users through word-of-mouth [16]. Previous research has shown how search trends and Wikipedia views are related to price changes [17] and to the speculative and monetary aspects of Bitcoin [18], leading to dynamics that combine search interest, user adoption, word-of-mouth and prices [16].

### 1.1 Contributions of this article

Based on established principles of time-series analysis and financial trading, we present a framework to derive general knowledge from multidimensional data on social and economic aspects of a market. We apply a general statistical model to detect temporal patterns in the co-movement of price and other signals. Those patterns are tested through a method robust to the empirical properties of the analysed data, formulating concise principles on which signals precede market movements. We combine those principles to produce tractable trading strategies, which we evaluate over a leave-out sample of the data, quantifying their profitability. Our approach, rather than focusing on improving a particular method, takes a multidisciplinary stance in which we combine principles from social psychology and economics with methods from information retrieval, time-series analysis and computational finance.

We apply our framework to the Bitcoin ecosystem, monitoring the digital traces of Bitcoin users with daily resolution. We combine *economic signals* related to market growth, trading volume, and use of Bitcoin as means of exchange, with *social signals* including search volumes, word-of-mouth levels, emotional valence and opinion polarization about Bitcoin. Our results reveal which signals precede changes of Bitcoin prices, a knowledge that we use to design algorithmic trading strategies. We evaluate the power of our strategies through backtesting data-driven simulations, comparing returns with technical analysis strategies. As a consequence, we test the hypothesis that social media sentiment predicts financial returns in the Bitcoin ecosystem.

### 1.2 Social signals in finance

Understanding the role of social signals in finance not only has the potential to generate significant profits, but also has scientific relevance as a research question [19]. Two different research approaches give insights to this question: one is the *statistical analysis* of social and financial signals in order to test the existence of temporal correlations that lead financial markets. The second one applies these signals in *prediction scenarios*, measuring their accuracy as a validation of the underlying behaviour of the system, but not necessarily of their profitability. The statistical analysis of search engine data reveals that search trends can predict trading volumes of individual stocks [20]. In addition, stock prices in S&P 500 are correlated with tweet volumes [21], but the applicability of these patterns into trading strategies is yet to be evaluated.

Sentiment in social media is closely related to socio-economic phenomena, including public opinion [22]. This motivates the application of sentiment indicators in the statistical analysis of financial data. Early works on the sentiment in specialized forums gave negative results about their impact on returns [23]. Further research showed that emotions in private instant messaging between workers of a trading company precede stages of market volatility [24]. The expression of anxiety in publicly accessible data from general blogs precedes trading peaks and price drops in the S&P 500 [25], and sentiment in Twitter can be used to predict movements in large-scale stock indices [26]. It is important to note that, to date, there is no evidence that such sentiment-based predictions produce significant returns on investment [19].

### 1.3 Online polarization

While most previous works on sentiment in financial markets focus on dimensions of valence or mood, the collective phenomenon of polarization of opinions is often overlooked. The emergence of polarization in a society gives early warnings on political and economic phenomena: polarization in social networks of Swiss politicians precedes controversial elections [27], and polarization patterns in the Eurovision Song Contest appear before states of distrust in the European economy [28]. With respect to financial markets, speculation theories point to the role of diverse beliefs in financial transactions [29], leading to the hypothesis that polarization and disagreement influence trading volumes and prices [30]. In this line, the empirical analysis of polarization in stock message boards shows that states of disagreement lead to increased volatility [31].

### 1.4 The missing link

To date, there is a significant knowledge gap between the analysis and application of social signals to trading scenarios. Findings from statistical analyses alone are not guaranteed to lead to profitable strategies at all [25]. For example, movements of the Dow Jones Industrial Average (DJIA) can be predicted with mass media sentiment [32] and Twitter mood [26], but to date no research has shown that such prediction methods can be profitable in trading scenarios. Similarly, the analysis of discussion patterns in specialized blogs predict returns of some technology companies [33], but it is still open to evaluate the potential returns of such a predictor. The application of methods that process arbitrarily large datasets lead to results difficult to apply, for example the predicting power of search volumes of the query *‘moon patrol’* [34] in backtesting over the DJIA [6]. Furthermore, analyses of Twitter discussions about companies can be applied in a portfolio strategy, yet its evaluation through backtesting leads to very moderate returns and their statistical significance is not assessed [35]. In addition, no previous research has proposed a prediction technique that derives significant returns on investment from online sentiment data [19]. Our research aims at closing the gap between these lines of research. To do so, we unify the statistical analysis and its application to design and evaluate trading strategies, based on tractable principles with potential impact in the finance community.

## 2. Trading strategy framework

**Figure 1.**
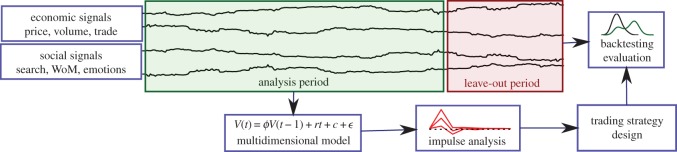
Framework for analysis of social and economic signals and trading strategy design and evaluation.

To design and evaluate trading strategies, we present a framework that uses a set of economic and social signals related to the agents of the market under scrutiny. Among those signals, the only required one is an economic signal of prices of an asset, namely a stock, currency, or tradable index. To understand profitability, we convert the price time-series *P*(*t*) into a return time series:

2.1 $$\mathrm{Ret}(t)=\frac{P(t)-P(t-1)}{P(t-1)},$$

which quantifies proportional changes in the price at every time step. The data on these signals are divided in an analysis period and a leave-out period, as depicted in figure 1. The division in these periods needs to allocate enough data in the leave-out sample to provide the testing power to assess the statistical significance of strategy profits. For daily trading, a leave-out period of about 1 year is usually sufficient, but this ultimately depends on the expected profitability and variance of the trading strategies.

### 2.1 Multidimensional analysis

The first step in our framework focuses on the analysis period, applying a multidimensional model of vector auto-regression (VAR) [36], which is commonly used in the analysis of multidimensional time series in finance [16,23,37]. A VAR models multidimensional linear relations with given lags, which in our analysis we set to 1 day. Thus, given the vector of signals *V* (*t*), we fit the equation

2.2 V(t)=ϕV(t−1)+r×t+c+ϵ,

where *ϕ* is a matrix of weights of the linear relations between variables, *r* is a deterministic trend vector, *c* is the vector of constant intercepts and *ϵ* is a vector of uncorrelated errors. While more advanced models can be considered, including longer lags and nonlinear terms, we choose the VAR model of lag 1 for its general character and its proved power to reveal patterns in finance [16,23]. More complex models might have higher power to reveal nuance patterns, but at the expense of a loss of generality owing to the focus on particular systems.

We include all the time series in a single model to avoid the false positives associated with pairwise Granger tests. To ensure the correct application of the VAR model, we need to verify that our analysis is consistent with its fundamental assumptions: (i) that the elements of *V* (*t*) do not have a unit root, and (ii) that the error term *ϵ* has no temporal nor structural correlations. We verify the first set of assumptions on the properties of *V* (*t*) by applying a set of tests and transformations prior to the application of the VAR model. We ensure that our conclusions are robust to the second set of assumptions by correcting for correlations in the noise term, as explained in the Material and methods section.

### 2.2 Impulse analysis

The VAR weights *ϕ* are only informative when there are no correlations in the error term *ϵ* of equation (2.2), which is usually not the case in practice. To extract stylized facts that can be used in the design of trading strategies, we perform an impulse analysis by measuring impulse response functions (IRF) [38] while correcting for correlations in the empirical error. This method simulates the system dynamics when it receives a shock in one of the variables, applying the VAR dynamics of equation (2.2) to reproduce the changes in the rest of the variables through time. By recording the changes in each variable, we can estimate the total size and the timespan of the perturbation produced by the shock. In essence, the IRF method creates a computational equivalent of the system under scrutiny, to test its reaction to exogenous impulses in each of its elements.

### 2.3 Trading strategy design and evaluation

The output of the impulse analysis step, shown in figure 1, is a set of patterns of Granger-type ‘causation’, i.e. it tests the null hypothesis of the absence of temporal correlations among the variables. We use these patterns as stylized facts that indicate which variables precede changes in price returns. For example, if variable *Y* (*t*) has a significant impact on Ret(*t*) in the impulse analysis, we will include *Y* (*t*) in our trading strategy design with sign *s*_*Y*_, which takes the value 1 if the response of Ret(*t*) to *Y* (*t*) was positive, and −1 otherwise. Thus, a predictor based on *Y* (*t*) would be

2.3 $$\mathrm{sign}(\mathrm{Ret}(t+1))=\mathrm{sign}(s_{Y}\times (Y(t)-Y(t-1))).$$

This way, we predict increases (decreases) in price between time *t* and *t*+1 if signals with positive responses increase (decrease) between time *t*−1 and *t*, and vice versa for signals with negative responses. Since our multidimensional analysis is robust to confounds between multiple time series, the findings of impulse analysis can be integrated in a *Combined* strategy based on a voting mechanism. The *Combined* strategy applies the other predictors and formulates a prediction corresponding to the sign of the sum of their outputs, i.e. the majority vote.

We evaluate the profitability of the designed strategies in comparison to the benchmark of standard strategies, based on the backtesting over the leave-out sample as indicated in figure 1. For each strategy, we make a data-driven simulation of a trader following that strategy, and we record the profits of that trader on a daily basis. Details on the computational simulation of financial traders can be found in the Material and methods section.

### 2.4 Bitcoin social economic and signals

**Figure 2.**
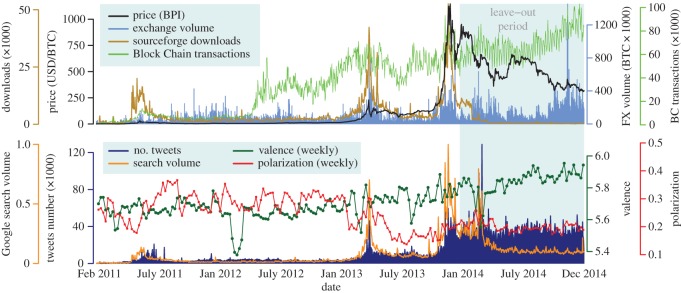
Time series of data volumes in the Bitcoin ecosystem. Interactive version: www.sg.ethz.ch/btc.

We apply our approach to the case of trading Bitcoin based on social and economic signals of the Bitcoin ecosystem. We set up a system that monitors different data sources, retrieving data in real time in combination with historical time series. The data volumes recorded during our study period of almost 4 years are shown in figure 2, and can be interactively browsed in our online visualization (www.sg.ethz.ch/btc). The signals we measure, explained more in detail in the Material and methods section include economic signals of price *P*(*t*) and returns Ret(*t*), trading volume in a wide range of Bitcoin exchange markets FX_Vol_(*t*). Furthermore, we measure the economic signal of transaction volume in the Block Chain BC_Tra_(*t*), which measures the volume of usage of Bitcoin as a currency, and the amount of downloads of the most important Bitcoin client Dwn(*t*) as a measure of growth in adoption of the Bitcoin technology. The social signals we measure are the level of search volume in Google for the term ‘bitcoin’ *S*(*t*), the word-of-mouth level as measured by the amount of *tweets* containing Bitcoin-related terms *T*_*N*_(*t*), and the emotional valence *T*_Val_(*t*) and opinion polarization *T*_Pol_(*t*) expressed in those tweets using lexicon-based approaches from psycholinguistics [39,40] (more details in Material and methods). All these signals are shown in figure 2, illustrating the large oscillations of price and other signals related to Bitcoin.

## 3. Results

### 3.1 Data-driven Bitcoin trading strategy design

For our statistical analysis, we include all the data up to 1 January 2014, covering almost 3 years. After applying stationarity tests, we conclude that the time series of price returns Ret(*t*) can be assumed to be stationary, as well as the first differences of the other seven signals (details on the stationarity test results can be browsed in www.sg.ethz.ch/btc and in the electronic supplementary material). As a consequence, we define our variable vector as

$$V(t)=[\mathrm{Ret}(t),\Delta {\mathrm{FX}}_{\mathrm{Vol}}(t),\Delta {\mathrm{BC}}_{\mathrm{Tra}}(t),\Delta \mathrm{Dwn}(t),\Delta S(t),\Delta T_{N}(t),\Delta T_{\mathrm{Val}}(t),\Delta T_{\mathrm{Pol}}(t)],$$

composing the input to the multivariate analysis of our framework. We fit a VAR as explained in Material and methods over the analysis period. We compute IRF for all pairs of variables, all results including VAR estimates and IRF values can be browsed in www.sg.ethz.ch/btc and in the electronic supplementary material. Here, we comment on the most relevant results, which serve as input for our trading strategy design.

**Figure 3.**
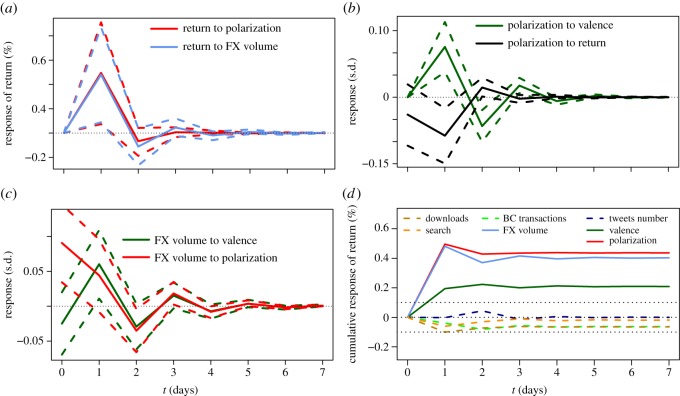
Results of IRF analysis. (*a*) IRF of return to shocks in Twitter polarization and exchange volume, (*b*) of Twitter polarization to shocks in return and Twitter valence, and (*c*) of exchange volume to shocks in Twitter valence and polarization (right). Solid lines show responses, dashed lines show 95% confidence intervals. (*d*) Cumulative IRF of price return to changes in the other signals. Dashed lines indicate responses below the 0.1% level.

Figure 3*a* shows the IRF of returns to shocks in polarization and volume in exchange markets, where the response is measured in return percentages. Both polarization and exchange volume have significantly positive influences in price returns 1 day after the shock, decreasing rapidly afterwards. The increase of returns with polarization is consistent with the hypothesis that disagreement fuels trading in speculative scenarios [30,31], where information asymmetries fuel price bubbles. Exchange volume also increases with polarization, as shown in figure 3*c*, but the relationship is instantaneous rather than lagged as in the case of returns.

Figure 3*b* shows the response of polarization in Twitter to shocks in returns and valence. The negative effect of polarization shows that price drops lead to increases in polarization, signalling the disagreement in the Bitcoin community owing to price crashes. The pattern linking valence to polarization is relevant, revealing that periods with increasing positivity in expression precede stages of higher polarization. The role of valence can further be observed in the IRF of exchange volumes in figure 3*c*, in which valence has a significant effect. The combination of patterns of increasing polarization and exchange volume following stages of increasing valence show the relevance of valence in price returns, in addition to the effects of polarization and exchange volume.

We further validated these results in two ways. First, we fit a VAR with lags longer than a day, selecting the optimal lag that optimizes the Bayesian information criterion. We found that a lag of 2 is optimal, but the results of the fits and IRF analysis did not qualitatively change (see the electronic supplementary material). Second, we performed a Monte Carlo test, computing the IRF for time series with randomized permutations of the values. The results of these permutation tests are consistent with the above results, as reported in the electronic supplementary material, showing the robustness of our approach.

### 3.2 Turning analysis into strategy

We summarize the above findings as stylized facts that can drive the decisions of an algorithmic trader. We focus closer on the role of each signal into returns, by computing the cumulative changes given by the IRF analysis. This way, we can identify which signals show a sizable pattern that precedes changes in returns, and filter out those that are not significant or can be explained as confounds of the others. Figure 3*d* shows the results, measuring the cumulative change in return percentage when each one of the other signals receives a shock of size 1 s.d. The three signals with effects above the 0.1% level are polarization, valence and exchange volume, reaching effects up to 0.5% in 1 day that prevail through time. Note that this is a relatively large value, because trading results in multiplicative returns. Such effect sizes have strong potential impact on the profitability of trading strategies over long time periods. This allows us to discard the rest of the signals, feeding into our trading strategy design by producing four strategies: three strategies of positive sign, *Valence*, *Polarization* and *FXVolume*, and a fourth *Combined* strategy determined by a voting mechanism as explained in the Trading strategy framework section.

### 3.3 Bitcoin strategy evaluation

To evaluate the profitability of our four strategies, we set up a benchmark against random strategies and technical strategies, using the actual exchange rate of BTC for USD in bitfinex.com as well as the Bitcoin Price Index (BPI; see www.sg.ethz.ch/btc for results with BPI). Random strategies sample a random number with 0 mean at every time *t* and formulate a prediction based on the sign of the random number. Among technical strategies, the simplest is *Buy and Hold*, which simply buys BTC with the initial capital at time *t*=1, selling it only once at the time when profits are evaluated. The technical strategies we use are a benchmark of simple standard predictions [5]: (i) the *Momentum* strategy, which predicts that price changes at time *t*+1 will be the same as at time *t*, (ii) the up and down persistency strategy *UPD*, which predicts that price increases at time *t* are followed by decreases at time *t*+1, and vice versa, and (iii) the relative strength index strategy *RSI*, which computes an additional time series of ratios of return sign frequencies over a rolling window of 5 days, and predicts price changes based on reversals of this time series (more details in [5]).

**Figure 4.**
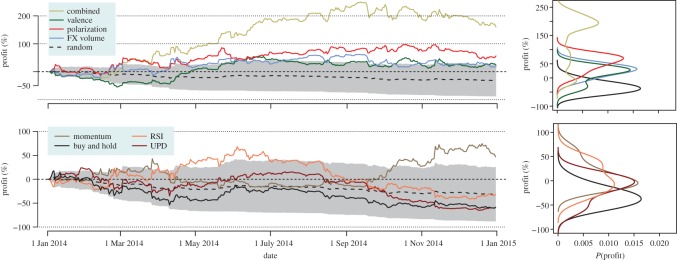
Profits of trading strategies. Left: time series of profit for our strategies (top) and technical strategies (bottom). Shaded areas show 1 s.d. of the random strategy. Interactive version: www.sg.ethz.ch/btc. Right: kernel density plots of the profit of each strategy (*bandwidth*=15%).

The simulation of each strategy produces a time series of profits:

3.1 $$\mathrm{Profit}(t)=\frac{C(t)-C(0)}{C(0)}\times 100,$$

where *C*(*t*) is the capital of the trader at time *t* and *C*(0) is the initial investment capital. Figure 4 shows the time series of profits for our four strategies and the technical strategies. In addition, we compute the profit of *Buy and Hold*, and the results of the simulation of 10 000 random traders. The *Valence*, *Polarization* and *Combined* strategies clearly perform better than a random trader, while the *FXVolume* is not very far from the result of random traders. Among the technical strategies, only *RSI* and *Momentum* are able to eventually reach beyond the outcome of random traders, but are still clearly outperformed by the *Polarization* and *Combined* strategies.

The stopping time of the simulation of a trading strategy is given by our data, but a variety of factors might trigger a trader to stop trading earlier in a real scenario [3]. For that reason, we explore the distribution of profits of each strategy, assuming that the trading stops at any arbitrary point of our backtesting period. Thus, for each strategy, we have a set of profit values, one for each possible trading end date. The right panel of figure 4 shows the kernel density plots of the distributions of profits for each strategy. It can be appreciated that the most profitable strategy is *Combined*, followed by *Polarization* and then *Valence* and *RSI*. We quantitatively assessed this result, through Wilcoxon tests [41] over the distributions of profits (more details in the electronic supplementary material), confirming the observation that the most profitable strategies are *Combined*, and *Polarization*. More precisely, the *Combined* strategy gives profits beyond 100% for most of the time during the trading period.

While surveying cumulative returns is illustrative of the performance of the strategies, the multiplicative nature of cumulative returns overweights early positions and is biased towards the beginning of the evaluation period. To properly evaluate trading strategies, we calculated the Sharpe ratio [42], measuring risk-corrected profits as: *SR*=(*μ*_R_−*R*_f_)/*σ*_R_, where *μ*_R_ and *σ*_R_ are the mean and standard deviation of the daily rate of return of a strategy *R*(*t*)=(*C*(*t*)−*C*(*t*−1))/*C*(*t*−1). *R*_f_ is the ‘risk- free’ return rate of a theoretical investment that would give certain profit under no risk at all, which is often estimated as the interest rate of high-quality sovereign bonds. At the time of writing, some European sovereign bonds are giving interest rates close to zero or even negative [43], which motivates our conservative choice of *R*_f_=0. The value of SR is calculated in annualized units, taking into account that Bitcoin can be traded 365 days a year.

Table 1 reports the Sharpe ratio SR and the mean daily return *μ*_R_ for all strategies, as well as for the *DJIA* and the average of 10 000 random traders. The Sharpe ratio analysis is consistent with the results of the cumulative returns analysis, showing that the *Combined* strategy provides the highest returns, with the best SR value above 1.75 and with daily returns above 0.3% per day. The profitability of these strategies illustrate how social media sentiment can produce positive returns on investment, especially when including polarization measures beyond the trivial quantification of valence or mood.

**Table 1.** Sharpe ratios and mean daily returns of strategies.

	Combined	Polarization	Valence	FXVolume	Buy and Hold
SR	1.7653	1.0120	0.6410	0.5738	−0.7741
*μ*_R_	0.3229	0.1779	0.1183	0.1082	−0.1635

	Momentum	UPD	RSI	DJIA	random
SR	0.9146	−0.8990	−0.1772	0.7995	−1.6590
*μ*_R_	0.1625	−0.1736	−0.0346	0.0345	−0.0963

### 3.4 Costs and risks of the Combined strategy

**Figure 5.**
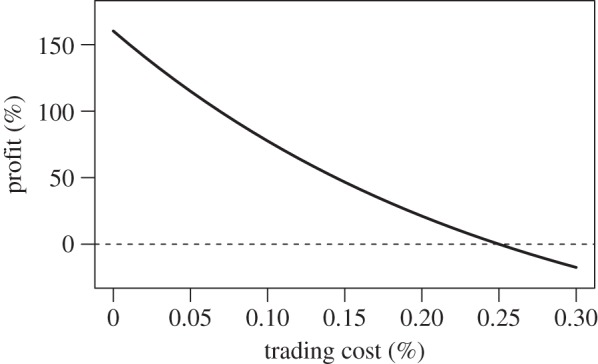
Final profit of the *Combined* strategy versus trading cost.

To better understand the possible weaknesses of the *Combined* strategy, we run a series of tests to evaluate the role of trading costs and additional risks. Trading Bitcoin in an online market usually comes at a cost, which often depends on the activity and the traded capital. These trading costs should not be confused with the transaction fees in the Block Chain [9], which do not depend on the transacted cost and are not associated to any market of exchange to other currencies. Trading costs can potentially erode the profitability of trading strategies, especially if they require many movements. We simulated the same backtests for costs increasing from 0 to 0.3% of the exchanged capital, a value well above the maximum costs of major trading platforms [44]. As a simplification, we assume that buying, selling and borrowing costs are the same, yet their values might depend on the trading volume of a strategy [44]. Figure 5 shows the final profits of the *Combined* strategy, which decrease monotonically with trading costs. The strategy is still highly profitable for low costs, but for costs above 0.25% the strategy is not profitable any more. Furthermore, we repeat this analysis assuming the limitation that daily positions need to be forcefully closed at the end of each trading period (shown in the electronic supplementary material), finding a decrease in returns but that the strategy is still profitable for trading costs of 0.1%, a typically high cost of current exchange platforms [45].

In this application, the leave-out period is characterized by decreasing BTC prices. Thus, it is important to evaluate the role of each possible trading action: longing when BTC are bought to be sold later, and shorting when they are borrowed and traded as explained above. We repeat the backtesting of the *Combined* strategy allowing only short and only long positions, following the methodology of Preis *et al*. [6]. As reported more in detail in the electronic supplementary material, the only short strategy yields higher cumulative returns than the only long strategy, as expected from a period in which prices decrease steadily.

We test further properties of the behaviour of the *Combined* strategy in the leave-out period. The distribution of daily returns of the *Combined* strategy during the leave-out period follows a lognormal distribution, as tested through maximum-likelihood fits and Kolmogorov–Smirnov tests (see the electronic supplementary material). The time series of returns of this strategy is also not autocorrelated and can be considered stationary (see the electronic supplementary material for stationarity tests of daily returns). This additional analysis shows that the high profitability of the *Combined* strategy is not owing to risky correlations in the behaviour of the trading strategy.

## 4. Concluding remarks

Our work applies established methods of time-series analysis and computational finance to integrate the analysis, design, and evaluation of trading strategies and social and economic signals. We have shown that our approach successfully reveals temporal patterns in the Bitcoin ecosystem, in particular the relationship between price returns and the signals of exchange volume and Twitter valence and polarization. Our statistical analysis is robust to noise correlations and the finite nature of time series, providing a consistent set of results that we can apply to strategy design. We evaluated the profitability of our strategies through data-driven simulations of a computational model of a trader, showing that a strategy which combines valence, polarization and exchange volume can reach very high profits in less than a year. The added value of including polarization in our analysis constitutes evidence that collective factors of emotions and opinions have the potential to predict financial returns, beyond trivial macroscopic aggregates like average valence.

Our framework can be applied to other trading scenarios in which social signals are available, like in the case of company stock trading driven by sales data, news information and social media sentiment towards a company. The general nature of our methods are of special relevance for real trading scenarios, as the stylized facts we use to design strategies provide a tractable explanation for their mechanisms. This allows traders to understand and evaluate the principles of the algorithmic trading strategies designed in our framework. Such tractability is an advantage in comparison to more complex, nonlinear, or subsymbolic models that do not have straightforward interpretations. Nevertheless, improvements can be expected from the addition of longer time lags, higher frequency trading and real-time optimization approaches. Furthermore, the rules that drive our trading strategies do not require retraining or calibration during trading, and the social and economic signals we employ can be quantified during a day in order to have an instant trading decision ready at the beginning of the next day. Our application to Bitcoin trading is thus realistic, making use of shorting options and performing well under the typical trading costs of Bitcoin markets [44].

The application of our results should be taken with caution. Historical profit through backtesting do not necessarily predict future ones, and the information sources analysed here could be adopted by Bitcoin traders. Our evaluation goes as far as the representativity of the leave-out sample, and future research should evaluate the performance of our approach when prices rise and when traders are aware of the existence of our trading strategies. Financial markets are known to quickly absorb knowledge, as it happened with the inclusion of search trends data in stock trading [7]. It is also difficult to estimate the scalability of automatic trading strategies, as financial markets are complex adaptive systems that react to trades of large volume. Furthermore, systemic risk emerges from algorithmic trading, creating *flash crashes* owing to algorithmic resonance [8]. In addition, structural changes and additional risks in borrowing and lending Bitcoin for shorting can emerge when exchange markets close or governments regulate Bitcoin, changing the rules of the game in a way such that our trading strategies might not work anymore.

With our study, we have shown that it is possible to turn social signals into profit. This extends the range of typical business applications for social media data like viral marketing or user engagement. Specifically, our combination of statistical analysis and backtesting serves as a framework for future applications of social media data in algorithmic trading. It allows a robust validation of strategy profits and a clear understanding of the system dynamics behind these profits. The application of our framework to Bitcoin trading illustrates that (asymmetric) information and profit are two manifestations of the same thing, and how traders can apply these macroscopic information sources to derive large profits. We foresee that the applications of social signals to finance will reach far beyond Bitcoin, not only to make private profit but also to understand the dynamics of individual and collective decisions and emotions.

## 5. Material and methods

### 5.1 Stationarity tests

Before fitting the VAR model, we test the stationarity of each time series through two alternative tests: (i) the augmented Dickey Fuller (ADF) test [46], which has the null hypothesis that the tested time series is *non-stationary*, and (ii) the Kwiatkowski–Phillips–Schmidt–Shin (KPSS) test [47], with the null hypothesis that the time series is *stationary*. Under these two tests, it can be considered safe that a time series is stationary if it passes the ADF test with a *p*-value below 0.05 and does not pass the KPSS test, giving a *p*-value above 0.1 [16,38]. We first analyse the time series of levels of each signal *X*(*t*), applying the differentiation operator Δ*X*(*t*)=*X*(*t*)−*X*(*t*−1) until each time series is stationary. This step is inspired in the Box–Jenkins method of ARIMA time-series analysis [48], and it is usual to reach stationarity after first differences [16,38]. The stationary properties of these time series imply that their means and standard deviations are bound, allowing us to renormalize them through the *Z*-transformation *Z*(*t*)=(*X*(*t*)−*μ*_*X*_)/*σ*_*X*_, where *μ*_*X*_ and *σ*_*X*_ are the mean and standard deviation of each time series. This way, all time series have the same scale and variance, and their effects in statistical analysis can be compared.

### 5.2 Impulse response function analysis

In the impulse analysis, we correct for the correlations in *ϵ* in two ways. First, we apply orthogonalized impulses of unit covariance, creating a shock of 1 s.d. in a variable under the error correlations of the VAR [49]. Second, we apply bootstrapping on the resulting responses by producing surrogate time series from resampling the residuals [38]. This way, we numerically compute confidence intervals of the responses in a very strict way, avoiding false positives and taking into account the finite size of the analysis period. In our case, we create 10 000 bootstrap samples to estimate 95% confidence intervals of the responses. As a result, we simultaneously measure the dynamics of the system and test their statistical significance.

### 5.3 Trading based on predictions

During each timestep, the prediction function makes a forecast either based on equation (2.3) or based on the price time series for technical strategies. Positive predictions translate into *buy* decisions when the trader does not own the asset, and *hold* if it does. When the predictor takes value 0, no change is done and the previous position is imitated. Negative predictions translate into *sell* positions when the trader owns the asset or *short* when it does not own it. Shorting works as follows: traders can make profit from correct predictions of price drops even if they do not own the asset predicted to drop in price. This is implemented by borrowing the asset, selling it first and buying it later for a lower price. The limitation for borrowing is usually imposed on the amount of capital already held by the trader, and often incurs in additional trading costs and legal regulations [50]. The simulation of each strategy produces a time series of profits, allowing us to measure their profitability based on historical data.

Buy and sell orders have respective costs *c*_b_ and *c*_s_, which are proportional to the total traded capital. In our case, we assume all costs are equal *c*=*c*_b_=*c*_s_, leaving particular realizations of the costs as open for future research. We compute daily cumulative returns when trading stops at *t*+1, holding USD or selling BTC at the price of *t*+1. Our trading simulations have a limit on short selling set by the amount of capital held by the trader and assume that short selling needs to be instantly executed, i.e. short positions are limited to one iteration. In summary, the strategy we execute is a single-asset backtesting scenario in which 100% of the capital is invested at each time step and shorting is limited. The pseudocode of this simulation is shown in algorithm 1.


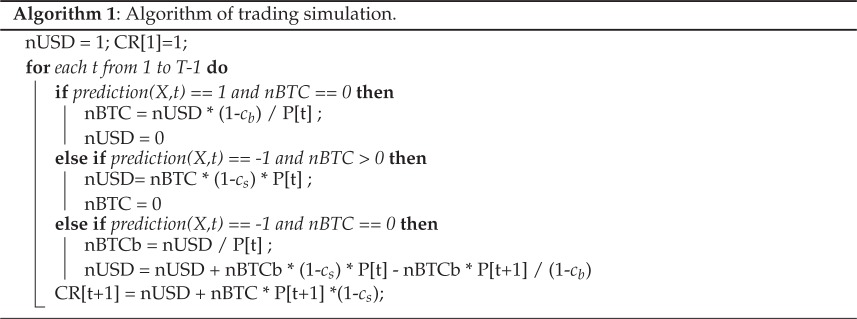


### 5.4 Economic signals from financial data

The establishment and bankruptcy of various Bitcoin exchange markets motivated the creation of the BPI [51]. The BPI combines a set of price indices from well-performing exchange marketplaces to provide a reference for BTC/USD exchange rates and is accepted as a standard measure of Bitcoin price in economics [18,52,53]. We use the daily closing prices of each day *t* at 23.59 GMT from coindesk.com, composing the time series of price *P*(*t*) from 1 February 2011 to 31 December 2014, shown in the top panel of figure 2. The BPI is not necessarily tradeable, and for that reason we evaluate our trading strategies with the actual exchange rate of BTC for USD in bitfinex.com, one of the largest markets reported in coindesk.com. We also retrieved the daily volume of BTC exchanged in 80 online markets for other currencies from bitcoincharts.com. Aggregating all these data sources, we compose an Internet-wide measurement of Foreign eXchange (FX) volume of BTC traded every day FX_Vol_(*t*), including more than 152 million BTC in exchange trades as we recorded in early 2015.

Every purchase of products and services in BTC leaves a trace in the *Block Chain*, the distributed ledger that records all transactions in the Bitcoin network. We construct a time series with the daily amount of Block Chain transactions BC_Tra_(*t*), as measured by blockchain.info every day at 18.15.05 UTC, which we approximate to 00.00 GMT of the next day. While some data are lost in this additional delay of a few hours, further research can provide more precise measurements up to the minute level using the raw information in the *Block Chain* itself as in [16]. This way, we include more than 55 million transactions in the studied period, measuring the overall activity of the system when using Bitcoin as means of exchange. In addition, we measure the growth of the Bitcoin market through its amount of adopters, using the operationalization of measuring the amount of downloads of the most popular Bitcoin client (http://sourceforge.net/projects/bitcoin) [16], daily binned in line with other time series. The resulting time series of downloads Dwn(*t*) is shown in the top panel of figure 2.

### 5.5 Social signals

We record the overall interest towards Bitcoin through information search, as quantified by the Google trends volume for the term ‘bitcoin’, *S*(*t*), as recorded in early 2015 and shown in the bottom panel of figure 2. We choose the search term ‘bitcoin’ instead of the ‘Bitcon - Currency’ topic, which was introduced as a functionality of Google Trends during our analysis period. While the topic approach can be more precise for demographics and motive analysis [12], we follow a homogeneous approach including only the term trend data that was available during the whole study period. It is important to note that Google Trends data is provided with an additional lag of 1 day and on the basis of Pacific Standard Time instead of GMT, adding almost another day of lag. While this is not an issue for the historical analysis, the evaluation of any trading strategy using *S*(*t*) needs to take into account this additional delay.

We track the attention of social media about Bitcoin in Twitter via the Topsy data service (http://topsy.com/). From the full track of data accessible by Topsy [54], we focus on tweets that contain Bitcoin terms as in previous research [16], finding a total of 19 578 671 Bitcoin-related tweets. The first social signal we extract from Twitter is the daily amount of unique tweets about Bitcoin *T*_*N*_(*t*) binned in 24 h windows starting at 00.00 GMT, measuring the level of word-of-mouth and attention towards Bitcoin and shown in the bottom panel of figure 2. We continue by measuring the collective emotional valence with respect to Bitcoin, as expressed through the text of Bitcoin-related tweets. Valence is considered the most important dimension of affect, quantifying the degree of pleasure or displeasure of an emotional experience [55]. The expression of valence through text is a common practice in psychological research, in which lexicon techniques are used to empirically measure emotions [56,57]. We measure the average daily valence of Bitcoin-related tweets through a state-of-the-art lexicon technique [40], which improves the previous ANEW lexicon method [56] with more than 13 000 valence-coded words. We compute the daily average Twitter valence about Bitcoin during day *t* in two steps: First, we measure the frequency of each term in the lexicon during that day, and second, we compute the average valence weighting each word by its frequency. This measurement matches more than 50 million valence-carrying tokens and produces the time series of Twitter valence *T*_Val_(*t*).

Our last social signal is opinion polarization, which builds up on measuring the semantic orientation of words into positive and negative evaluation terms [58]. We apply the LIWC psycholinguistics lexicon-based method [39] and expand its lexicon of stems into words by matching them against the most frequent English words of the Google Books dataset [59]. As a result, we consider 3463 positive and 4061 negative terms that appear as more than 8 million Twitter tokens. We compute the daily polarization of opinions in Twitter around the Bitcoin topic *T*_Pol_(*t*), calculating the geometric mean of the daily ratios of positive and negative words per Bitcoin-related tweet. Note that, instead of repeating a measurement of valence through two different lexica, we quantify polarization as a complementary dimension to emotional valence. This way, opinion polarization measures the simultaneous coexistence of positive and negative subjective content, rather than its overall orientation [23,58].

## Supplementary Material

Social Signals and Algorithmic Trading of Bitcoin - Supplementary Information

## Data accessibility

All data used for this article is publicly available through the corresponding Application Programming Interfaces. Processed data and scripts to reproduce these results are available at https://www.sg.ethz.ch/btc.
