# Supplementary material for: Social signals and algorithmic trading of Bitcoin
Source: R Soc Open Sci. 2015 Sep 23;2(9):150288. doi: 10.1098/rsos.150288 (PMC4593685; doi:10.1098/rsos.150288)
Supplement: Social Signals and Algorithmic Trading of Bitcoin - Supplementary Information [file rsos150288supp1.pdf]

Trading and social signals in the Bitcoin ecosystem

David Garcia and Frank Schweitzer

14.08.2015

Signals of the Bitcoin ecosystem

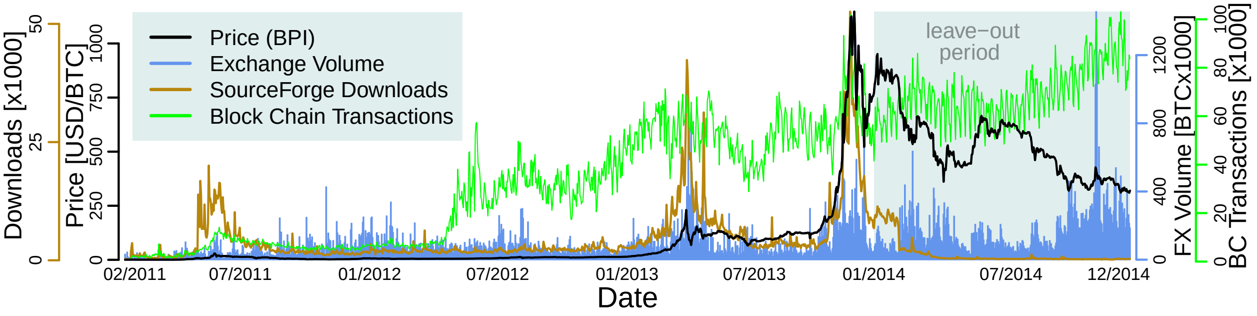

Figure 1: Time series of Bitcoin Price Index, FX Volume, amount of transactions in the Block Chain, and Bitcoin client downloads.

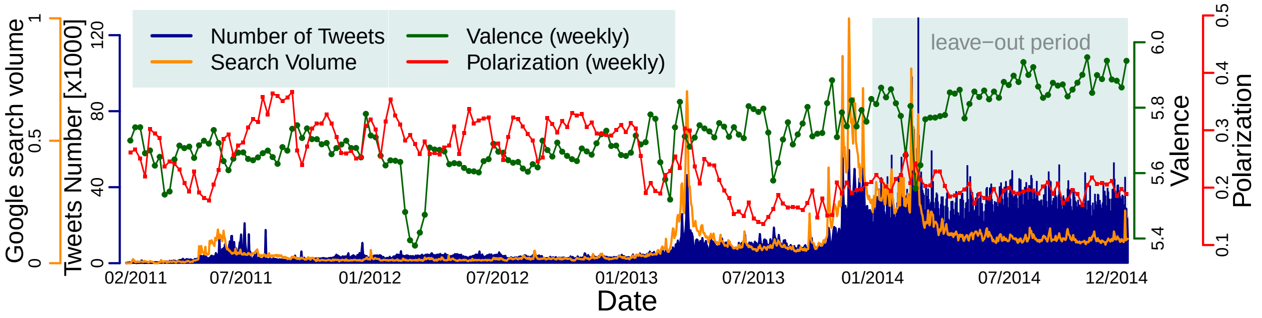

Figure 2: Time series of Bitcoin-related tweets, polarization, valence, and Google search volume.

FXVolume	BC Transactions	Downloads	Tweets
152439477	55210262	5449105	19578671

Table 1: Summary of dataset sizes.

Stationarity tests

signal	ADF	ADFpval	PP	PPpval	KSPP	KSPPpval	AR
Tweets	-7.7559748	0.0000000	-101.9368658	0.0100000	5.5212865	0.0100000	0.9135424
Polarization	-7.9836833	0.0000000	-158.1479700	0.0100000	4.6718462	0.0100000	0.8584331
Valence	-12.1639828	0.0000000	-493.8340651	0.0100000	2.8230069	0.0100000	0.6489829
Transactions	-8.2131394	0.0000000	-125.7243099	0.0100000	12.0026714	0.0100000	0.9999990
ExchangeVolume	-11.9483558	0.0000000	-475.7609587	0.0100000	2.6769065	0.0100000	0.6586341
Downloads	-5.8322548	0.0000037	-43.3071791	0.0100000	2.6960804	0.0100000	0.9992237
Search	-6.7051727	0.0000000	-41.1135810	0.0100000	3.9321100	0.0100000	0.9994038
BPI	-0.8243950	0.9619119	-3.5448747	0.9096929	4.3478948	0.0100000	0.9979938
Return	-22.6856583	0.0000000	-1044.5997512	0.0100000	0.1824587	0.1000000	0.0721432

Table 2: Stationarity tests for time series of levels and Return

signal	ADF	ADFpval	PP	PPpval	KSPP	KSPPpval	AR
Δ Tweets	-30.9634468	0.0000000	-833.1857606	0.0100000	0.0430182	0.1000000	-0.1391233
Δ Polarization	-31.5239392	0.0000000	-1023.3434173	0.0100000	0.0146565	0.1000000	-0.2593133
Δ Valence	-38.7433909	0.0000000	-1091.8257832	0.0100000	0.0107607	0.1000000	-0.3310941
Δ Transactions	-31.0196706	0.0000000	-883.7768285	0.0100000	0.0155640	0.1000000	-0.1592976
Δ ExchangeVolume	-32.3629574	0.0000000	-1109.4614927	0.0100000	0.0082475	0.1000000	-0.3398761
Δ Downloads	-25.7994780	0.0000000	-735.7927756	0.0100000	0.0154578	0.1000000	0.0783164
Δ Search	-27.9643026	0.0000000	-569.5286792	0.0100000	0.0265053	0.1000000	0.1654444

Table 3: Stationarity tests for time series of first order differences

Vector Auto-Regression

Response of Return

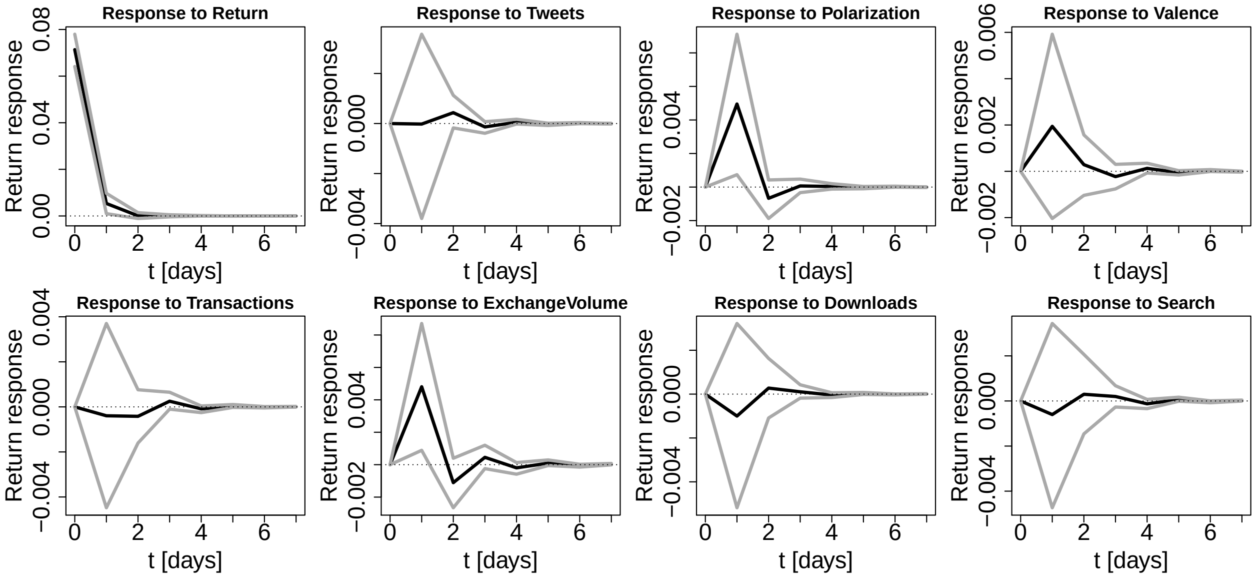

	VAR(1)	VAR(opt)
Return.l1	**0.09** (0.03)**	**0.09** (0.03)**
Tweets.l1	0.00 (0.00)	0.00 (0.01)
Polarization.l1	**0.00** (0.00)*	**0.00** (0.00)*
Valence.l1	0.00 (0.00)	0.00 (0.00)
Transactions.l1	−0.00 (0.00)	−0.00 (0.00)
ExchangeVolume.l1	**0.01** (0.00)*	**0.01** (0.00)**
Downloads.l1	−0.00 (0.00)	−0.00 (0.00)
Search.l1	−0.00 (0.00)	−0.00 (0.00)
const	0.01 (0.00)	0.01 (0.00)
trend	0.00 (0.00)	0.00 (0.00)
Return.l2		−0.01 (0.03)
Tweets.l2		0.00 (0.01)
Polarization.l2		−0.00 (0.00)
Valence.l2		0.00 (0.00)
Transactions.l2		−0.00 (0.00)
ExchangeVolume.l2		0.00 (0.00)
Downloads.l2		0.00 (0.00)
Search.l2		−0.00 (0.00)
R^2	0.02	0.02
Adj. R^2	0.01	0.00
Num. obs.	1065	1064
RMSE	0.07	0.07

$^{***}p < 0.001$, $^{**}p < 0.01$, $^{*}p < 0.05$

Table 4: Results for Return equation

Response of Number of tweets

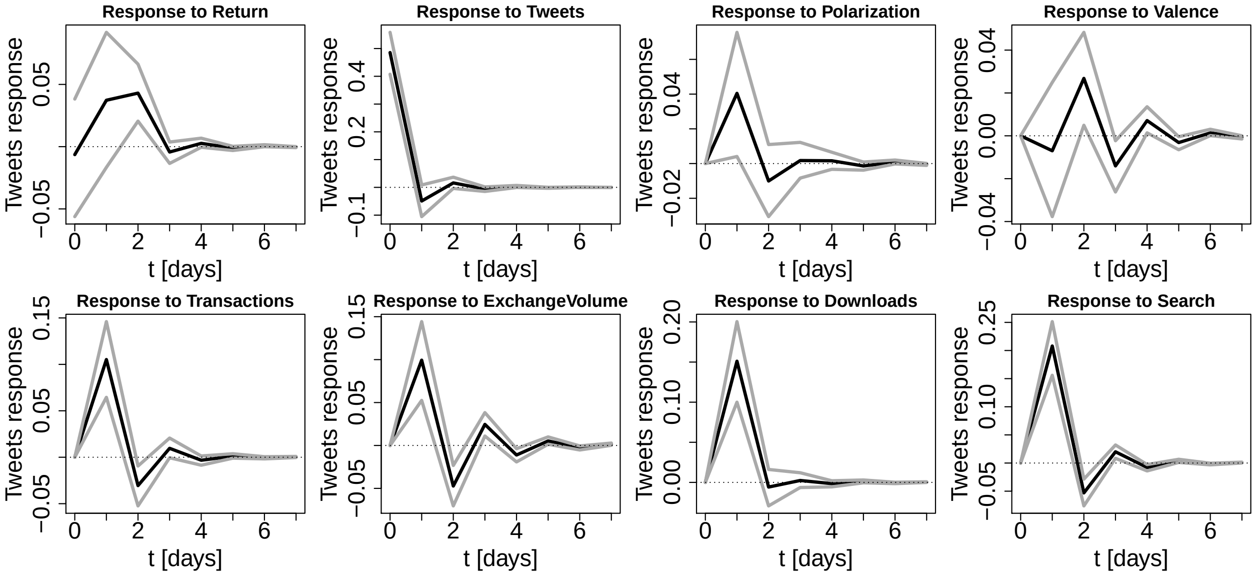

	VAR(1)	VAR(opt)
Return.l1	**0.64** (0.21)**	**0.66** (0.20)**
Tweets.l1	**−0.50** (0.03)***	**−0.55** (0.04)***
Polarization.l1	−0.01 (0.01)	−0.01 (0.01)
Valence.l1	−0.01 (0.01)	−0.01 (0.01)
Transactions.l1	**0.07** (0.02)***	**0.06** (0.02)***
ExchangeVolume.l1	0.03 (0.02)	**0.07** (0.02)***
Downloads.l1	0.02 (0.02)	0.02 (0.02)
Search.l1	**0.36** (0.03)***	**0.36** (0.02)***
const	−0.01 (0.03)	−0.01 (0.03)
trend	0.00 (0.00)	0.00 (0.00)
Return.l2		0.09 (0.21)
Tweets.l2		**−0.26** (0.03)***
Polarization.l2		0.01 (0.01)
Valence.l2		0.01 (0.01)
Transactions.l2		0.01 (0.02)
ExchangeVolume.l2		**0.08** (0.02)***
Downloads.l2		0.01 (0.02)
Search.l2		−0.04 (0.03)
R^2	0.30	0.38
Adj. R^2	0.30	0.37
Num. obs.	1065	1064
RMSE	0.49	0.46

$^{***}p < 0.001$, $^{**}p < 0.01$, $^{*}p < 0.05$

Table 5: Results for Tweets equation

Response of Polarization

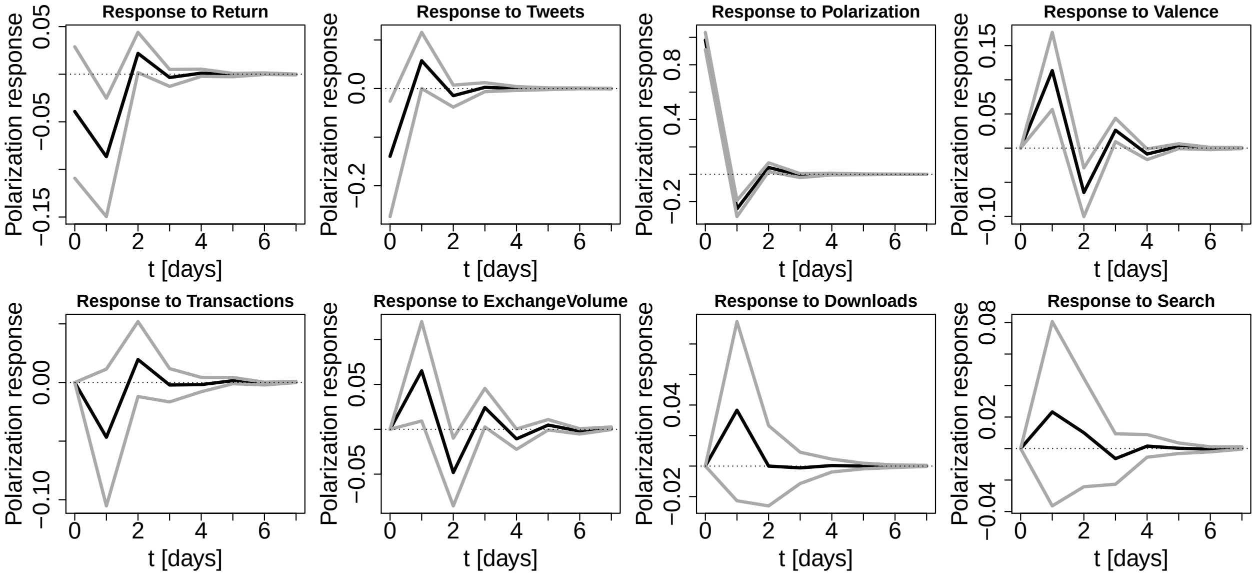

	VAR(1)	VAR(opt)
Return.l1	**−1.17** (0.44)**	**−1.06** (0.43)*
Tweets.l1	0.03 (0.07)	0.06 (0.08)
Polarization.l1	**−0.26** (0.03)***	**−0.30** (0.03)***
Valence.l1	**0.12** (0.03)***	**0.12** (0.03)***
Transactions.l1	**−0.08** (0.04)*	−0.07 (0.04)
ExchangeVolume.l1	0.07 (0.04)	0.06 (0.04)
Downloads.l1	0.02 (0.03)	0.02 (0.03)
Search.l1	0.04 (0.05)	0.02 (0.05)
const	0.01 (0.06)	0.01 (0.06)
trend	0.00 (0.00)	−0.00 (0.00)
Return.l2		0.30 (0.44)
Tweets.l2		−0.02 (0.07)
Polarization.l2		**−0.18** (0.03)***
Valence.l2		**0.08** (0.03)**
Transactions.l2		0.02 (0.04)
ExchangeVolume.l2		−0.07 (0.04)
Downloads.l2		0.00 (0.03)
Search.l2		0.04 (0.06)
R^2	0.10	0.14
Adj. R^2	0.09	0.13
Num. obs.	1065	1064
RMSE	0.99	0.97

$^{***}p < 0.001$, $^{**}p < 0.01$, $^{*}p < 0.05$

Table 6: Results for Polarization equation

Response of Valence

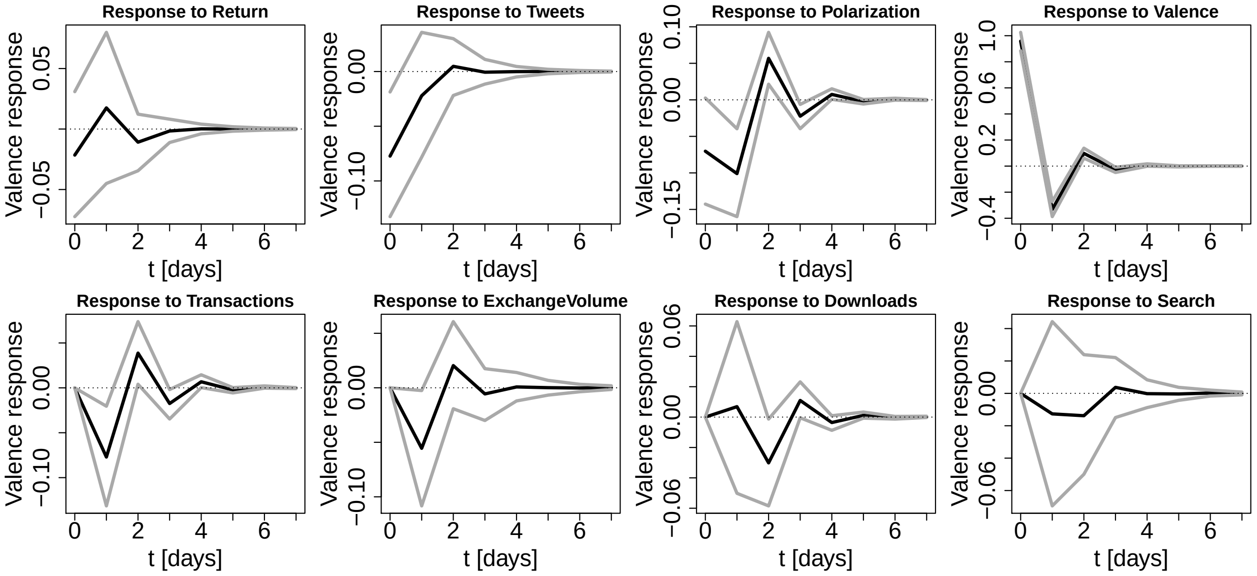

	VAR(1)	VAR(opt)
Return.l1	−0.09 (0.43)	0.04 (0.40)
Tweets.l1	−0.06 (0.06)	−0.09 (0.07)
Polarization.l1	**−0.11** (0.03)***	**−0.09** (0.03)**
Valence.l1	**−0.35** (0.03)***	**−0.47** (0.03)***
Transactions.l1	**−0.08** (0.04)*	**−0.08** (0.03)*
ExchangeVolume.l1	−0.07 (0.04)	−0.05 (0.04)
Downloads.l1	0.01 (0.03)	0.02 (0.03)
Search.l1	−0.02 (0.05)	−0.02 (0.05)
const	−0.01 (0.06)	−0.02 (0.06)
trend	0.00 (0.00)	0.00 (0.00)
Return.l2		−0.17 (0.41)
Tweets.l2		−0.03 (0.07)
Polarization.l2		−0.04 (0.03)
Valence.l2		**−0.36** (0.03)***
Transactions.l2		0.03 (0.03)
ExchangeVolume.l2		−0.02 (0.04)
Downloads.l2		0.02 (0.03)
Search.l2		−0.03 (0.05)
R^2	0.14	0.25
Adj. R^2	0.13	0.24
Num. obs.	1065	1064
RMSE	0.96	0.90

$^{***}p < 0.001$, $^{**}p < 0.01$, $^{*}p < 0.05$

Table 7: Results for Valence equation

Response of BC Transactions

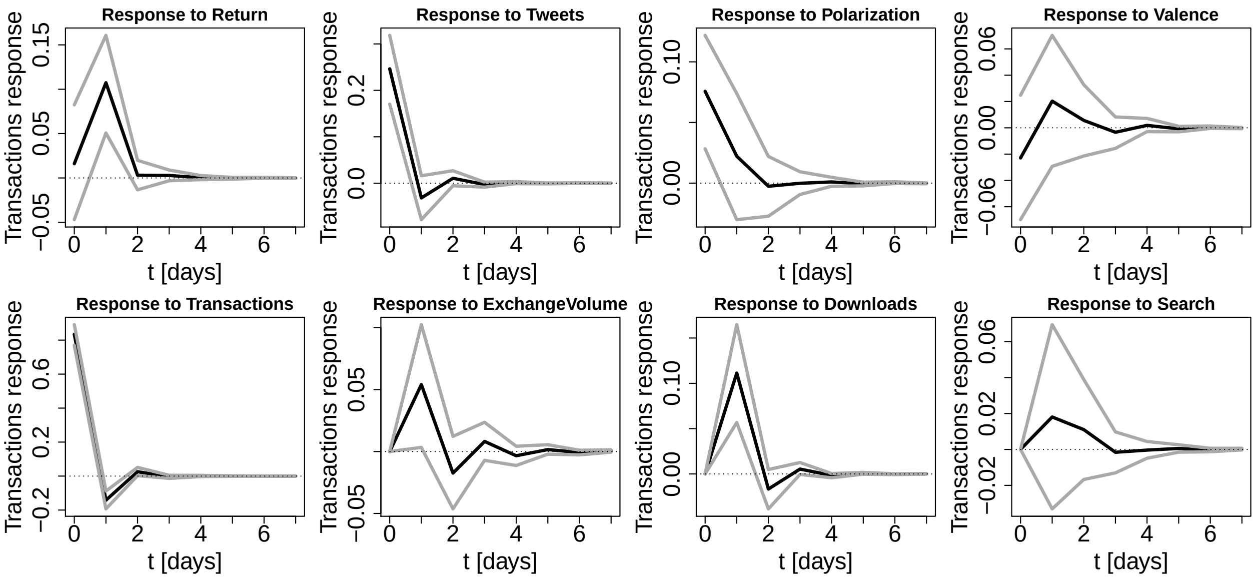

	VAR(1)	VAR(opt)
Return.l1	**1.52** (0.39)***	**1.51** (0.38)***
Tweets.l1	−0.06 (0.06)	−0.02 (0.07)
Polarization.l1	0.02 (0.03)	0.02 (0.03)
Valence.l1	0.01 (0.03)	0.01 (0.03)
Transactions.l1	**−0.19** (0.03)***	**−0.24** (0.03)***
ExchangeVolume.l1	0.04 (0.03)	0.05 (0.04)
Downloads.l1	**0.10** (0.03)**	**0.09** (0.03)**
Search.l1	0.03 (0.05)	0.05 (0.05)
const	−0.01 (0.05)	−0.01 (0.05)
trend	−0.00 (0.00)	−0.00 (0.00)
Return.l2		0.32 (0.38)
Tweets.l2		−0.08 (0.06)
Polarization.l2		0.02 (0.03)
Valence.l2		0.02 (0.03)
Transactions.l2		**−0.23** (0.03)***
ExchangeVolume.l2		0.03 (0.04)
Downloads.l2		−0.04 (0.03)
Search.l2		0.03 (0.05)
R^2	0.06	0.12
Adj. R^2	0.05	0.11
Num. obs.	1065	1064
RMSE	0.87	0.85

$^{***}p < 0.001$, $^{**}p < 0.01$, $^{*}p < 0.05$

Table 8: Results for Transactions equation

Response of FX Volume

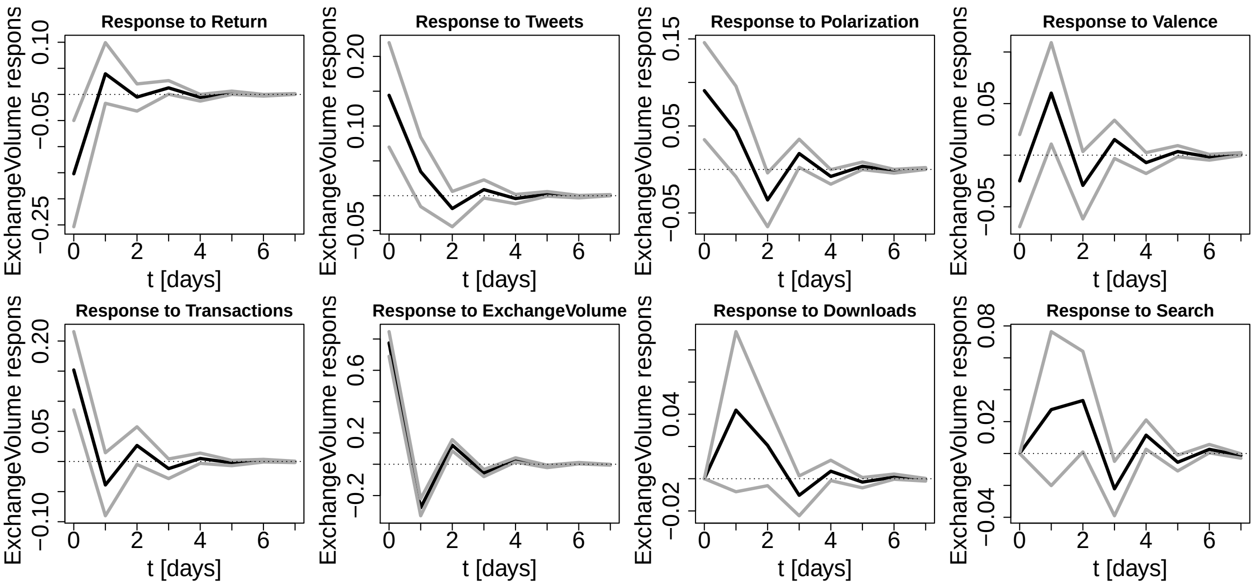

	VAR(1)	VAR(opt)
Return.l1	−0.19 (0.36)	−0.23 (0.35)
Tweets.l1	**0.15** (0.05)**	**0.17** (0.06)**
Polarization.l1	**0.08** (0.02)**	**0.09** (0.03)***
Valence.l1	**0.05** (0.02)*	**0.06** (0.03)*
Transactions.l1	0.01 (0.03)	0.03 (0.03)
ExchangeVolume.l1	−**0.38** (0.03)***	−**0.46** (0.03)***
Downloads.l1	0.02 (0.03)	0.02 (0.03)
Search.l1	0.05 (0.04)	0.04 (0.04)
const	0.01 (0.05)	0.01 (0.05)
trend	−0.00 (0.00)	−0.00 (0.00)
Return.l2		−0.12 (0.36)
Tweets.l2		−**0.16** (0.06)**
Polarization.l2		0.01 (0.03)
Valence.l2		**0.05** (0.03)*
Transactions.l2		0.03 (0.03)
ExchangeVolume.l2		−**0.26** (0.03)***
Downloads.l2		0.03 (0.03)
Search.l2		0.05 (0.05)
R^2	0.15	0.21
Adj. R^2	0.14	0.20
Num. obs.	1065	1064
RMSE	0.82	0.80

$^{***}p < 0.001$, $^{**}p < 0.01$, $^{*}p < 0.05$

Table 9: Results for ExchangeVolume equation

Response of Downloads

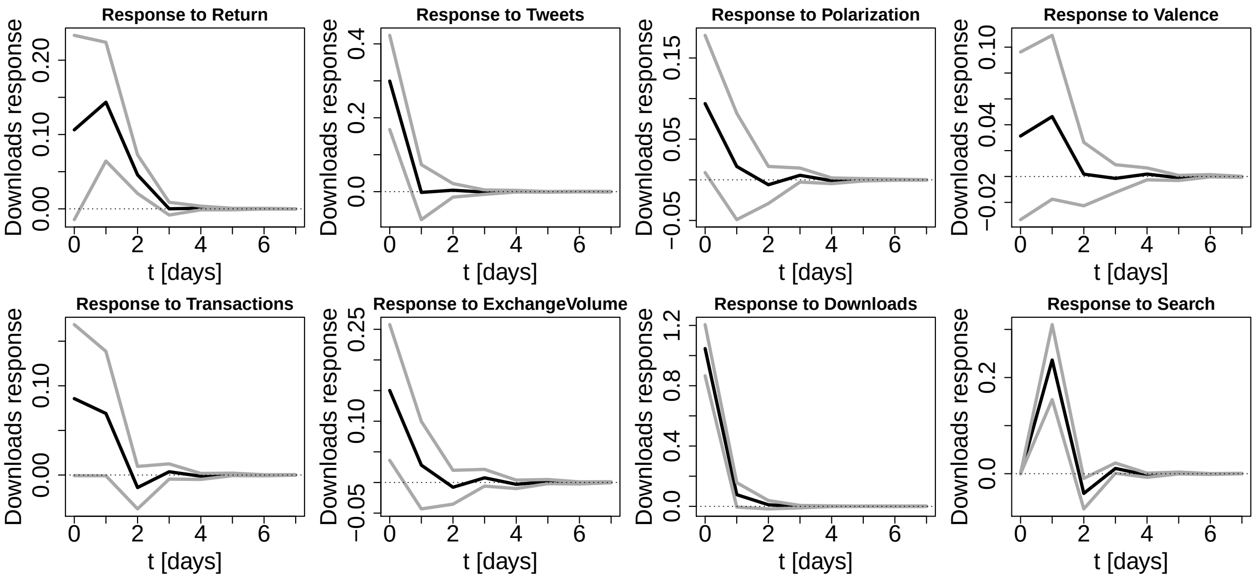

	VAR(1)	VAR(opt)
Return.l1	**2.13** (0.49)***	**2.30** (0.49)***
Tweets.l1	−**0.35** (0.07)***	−**0.35** (0.08)***
Polarization.l1	−0.02 (0.03)	−0.03 (0.03)
Valence.l1	0.04 (0.03)	0.03 (0.04)
Transactions.l1	0.04 (0.04)	0.06 (0.04)
ExchangeVolume.l1	−0.06 (0.04)	−0.02 (0.05)
Downloads.l1	−0.07 (0.04)	−**0.08** (0.04)*
Search.l1	**0.41** (0.06)***	**0.43** (0.06)***
const	−0.01 (0.07)	−0.01 (0.07)
trend	−0.00 (0.00)	−0.00 (0.00)
Return.l2		−0.43 (0.49)
Tweets.l2		−**0.17** (0.08)*
Polarization.l2		−**0.08** (0.03)*
Valence.l2		−0.02 (0.04)
Transactions.l2		0.07 (0.04)
ExchangeVolume.l2		0.07 (0.05)
Downloads.l2		−**0.15** (0.04)***
Search.l2		−0.01 (0.06)
R^2	0.07	0.11
Adj. R^2	0.06	0.10
Num. obs.	1065	1064
RMSE	1.11	1.09

$^{***}p < 0.001$, $^{**}p < 0.01$, $^{*}p < 0.05$

Table 10: Results for Downloads equation

Response of Search

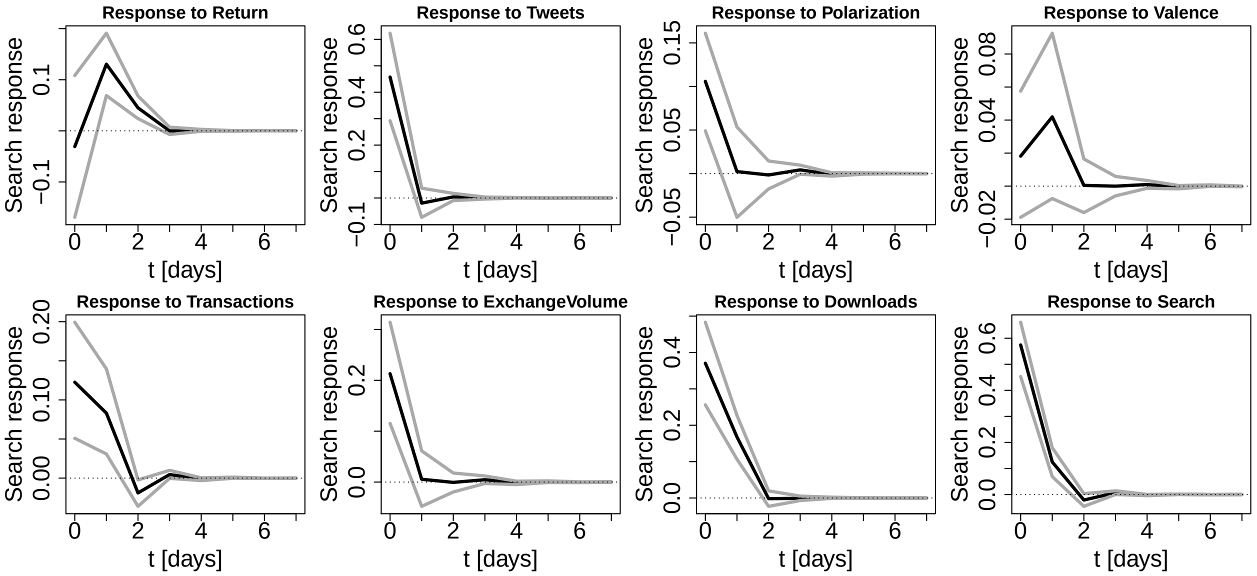

	VAR(1)	VAR(opt)
Return.l1	**1.60** (0.38)***	**1.65** (0.37)***
Tweets.l1	−**0.31** (0.06)***	−**0.19** (0.06)**
Polarization.l1	−0.03 (0.03)	−0.02 (0.03)
Valence.l1	0.04 (0.03)	0.03 (0.03)
Transactions.l1	**0.07** (0.03)*	0.06 (0.03)
ExchangeVolume.l1	−**0.07** (0.03)*	−0.03 (0.03)
Downloads.l1	**0.08** (0.03)**	**0.12** (0.03)***
Search.l1	**0.22** (0.05)***	**0.17** (0.04)***
const	−0.02 (0.05)	−0.02 (0.05)
trend	0.00 (0.00)	0.00 (0.00)
Return.l2		−0.12 (0.38)
Tweets.l2		−0.06 (0.06)
Polarization.l2		−0.04 (0.03)
Valence.l2		−0.00 (0.03)
Transactions.l2		0.05 (0.03)
ExchangeVolume.l2		**0.10** (0.03)**
Downloads.l2		**0.06** (0.03)*
Search.l2		−**0.33** (0.05)***
R^2	0.09	0.16
Adj. R^2	0.08	0.15
Num. obs.	1065	1064
RMSE	0.87	0.83

$^{***}p < 0.001$, $^{**}p < 0.01$, $^{*}p < 0.05$

Table 11: Results for Search equation

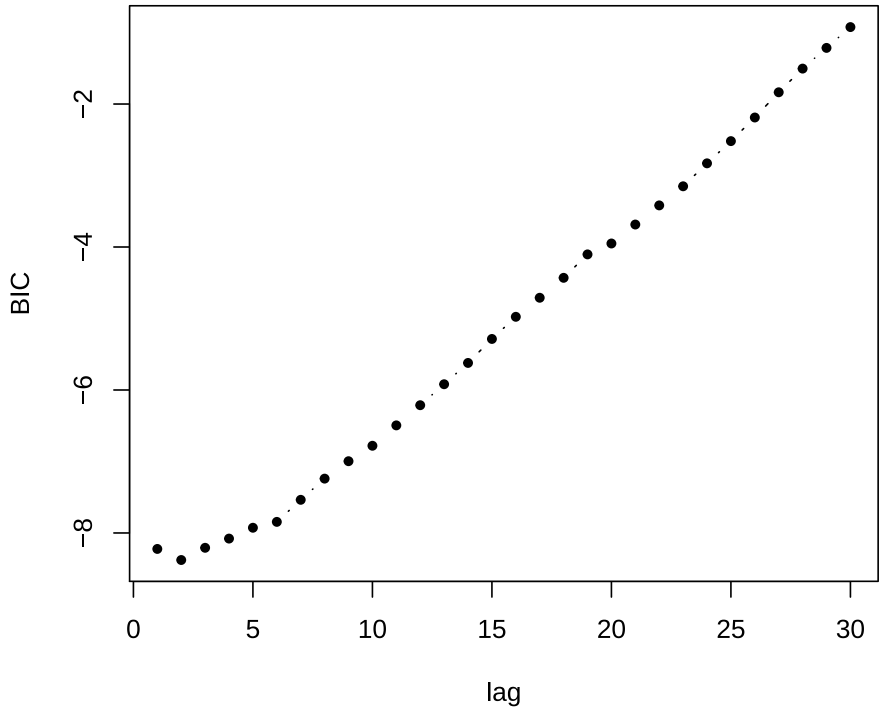

Figure 3: Bayesian Information Criterion for VAR fits of increasing lags.

Impulse Response Functions

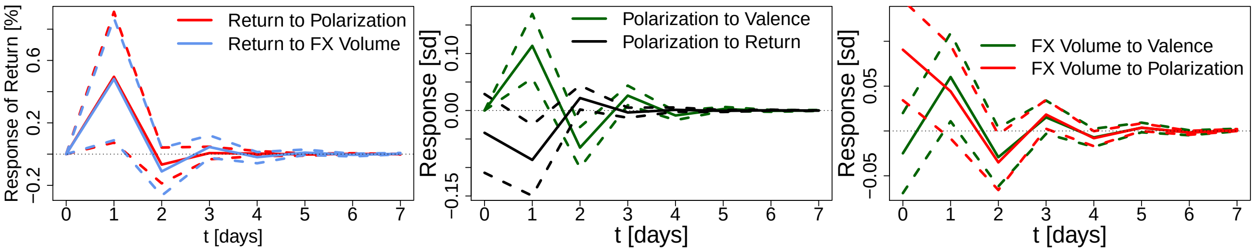

Figure 4: Significant impulse response functions of Return (left), Polarization (center) and FX Volume (right). Solid lines show HAC corrected estimates, dashed lines show bootstrapping 95% confidence intervals.

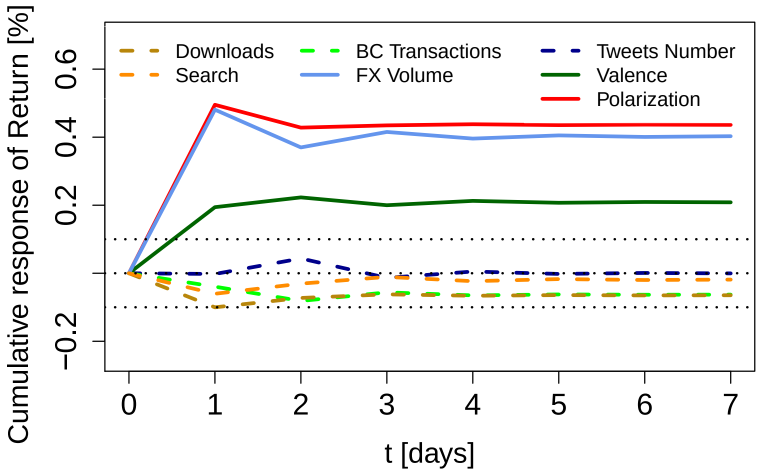

Figure 5: Cumulative impulse response functions of Return. Solid lines show sizable responses beyond +-0.1%.

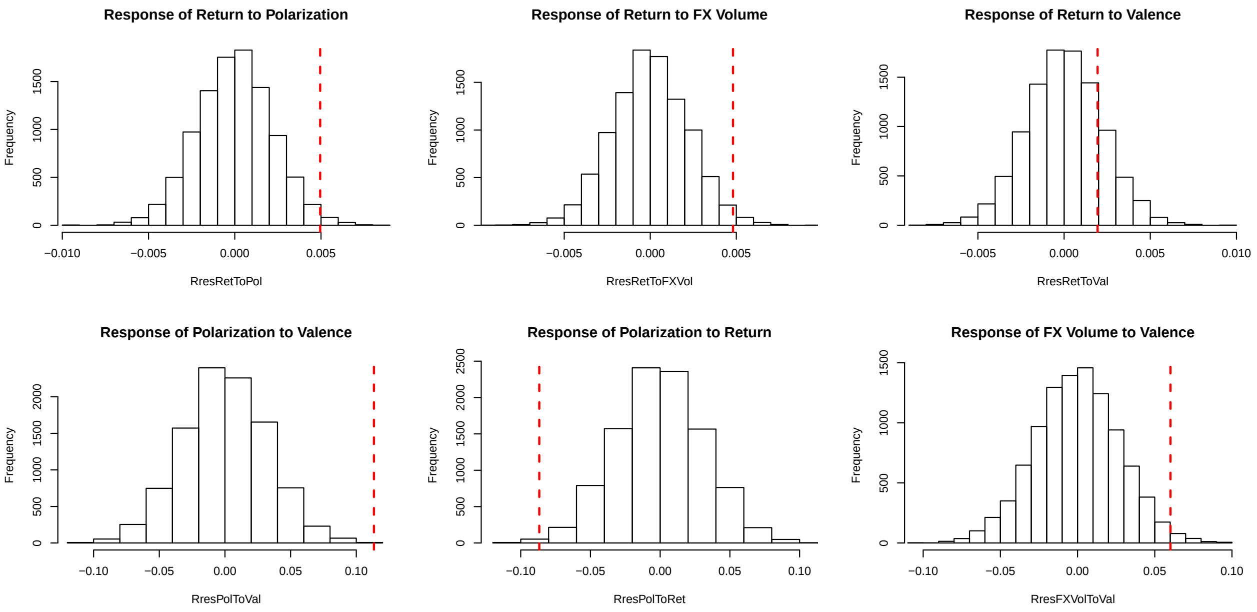

Figure 6: Results of Monte Carlo tests for the identified responses.

Trading Strategies Time Series

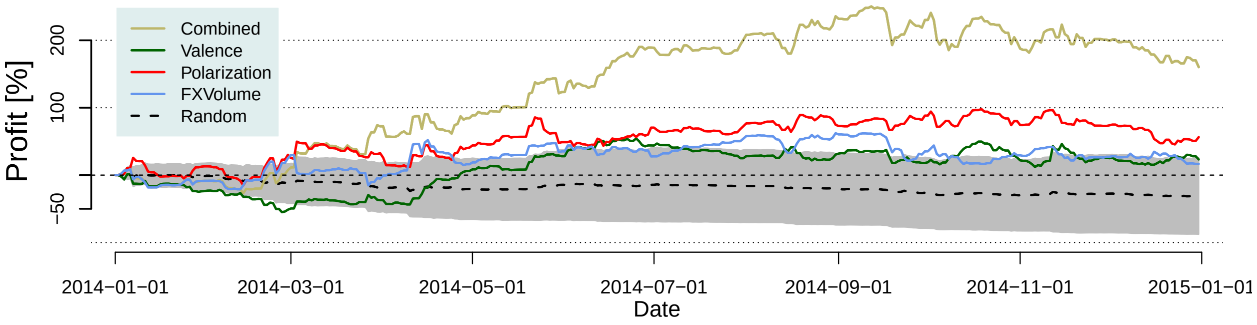

Figure 7: Time series of strategy cumulative returns. Shaded area shows one standard deviation around the random strategy.

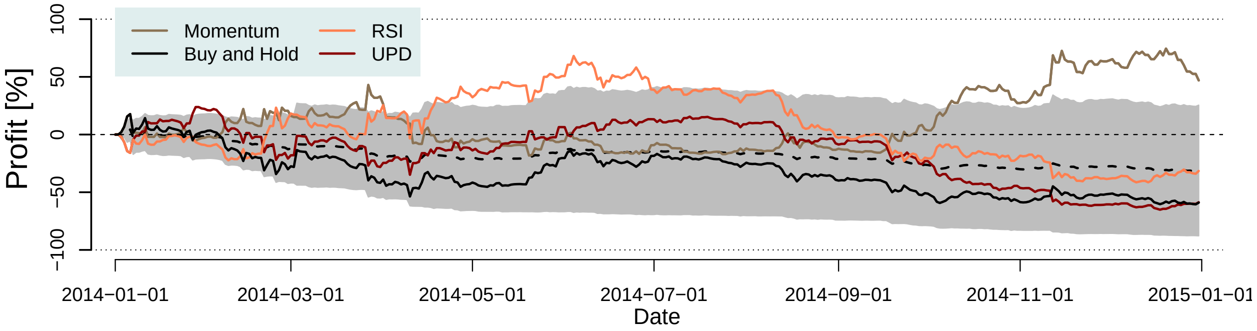

Figure 8: Time series of technical analysis cumulative returns.

Strategy Evaluation

	Combined	Buy&Hold	Pol	Val	FX Vol	MOM	UPD	RSI	DJIA	Rnd
SR	1.7653	-0.7741	1.0120	0.6410	0.5738	0.9146	-0.8990	-0.1772	0.7995	-1.6590
μ_R	0.3229	-0.1635	0.1779	0.1183	0.1082	0.1625	-0.1736	-0.0346	0.0345	-0.0963

Table 12: Sharpe Ratios and mean daily returns of strategies (%)

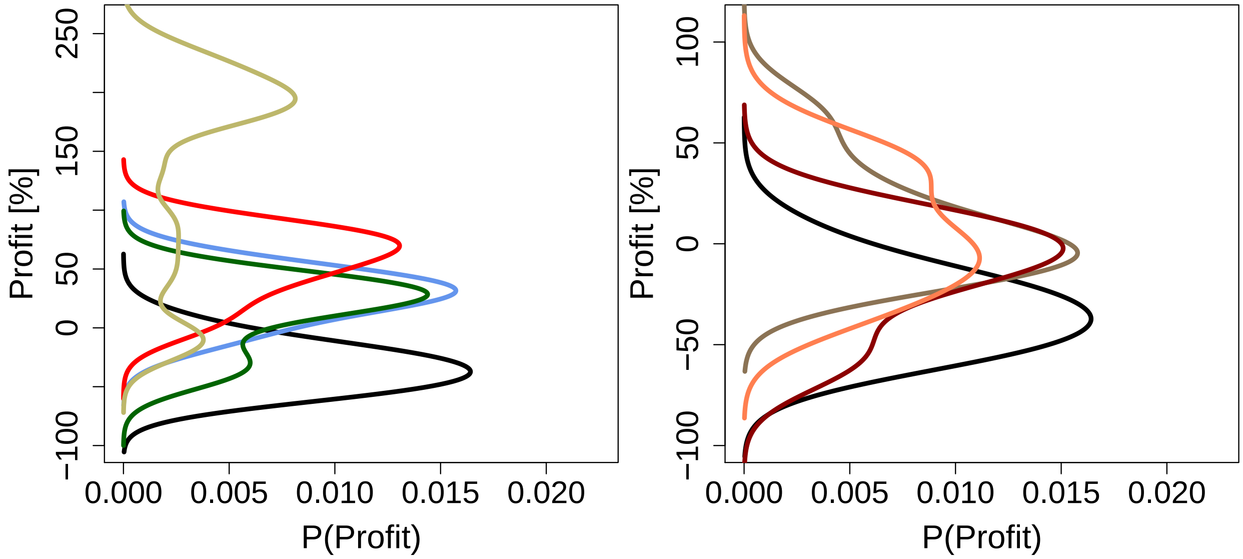

Figure 9: Kernel Density Plot of cumulative returns of data-driven and technical analysis strategies.

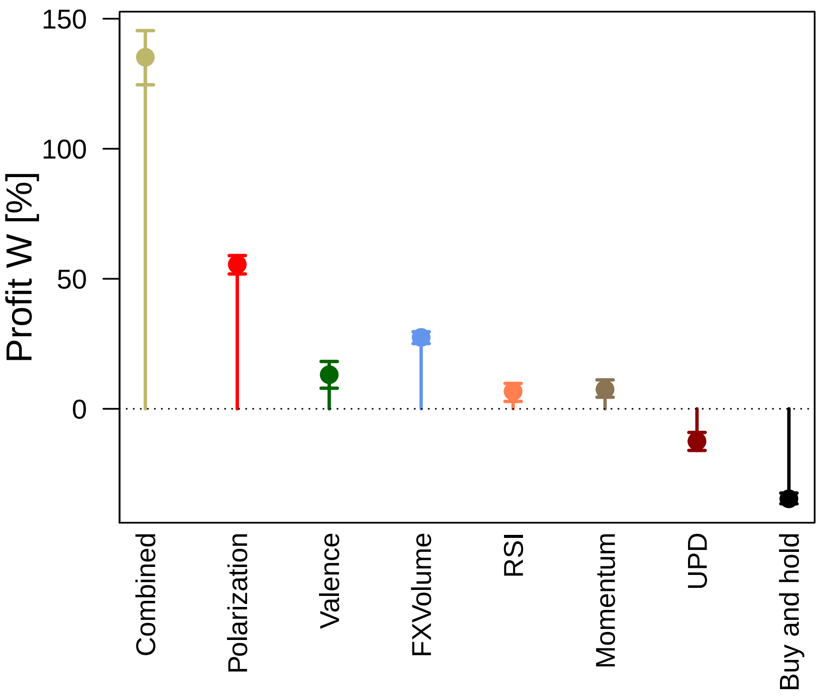

Figure 10: Wilcoxon estimates of cumulative returns.

Costs analysis

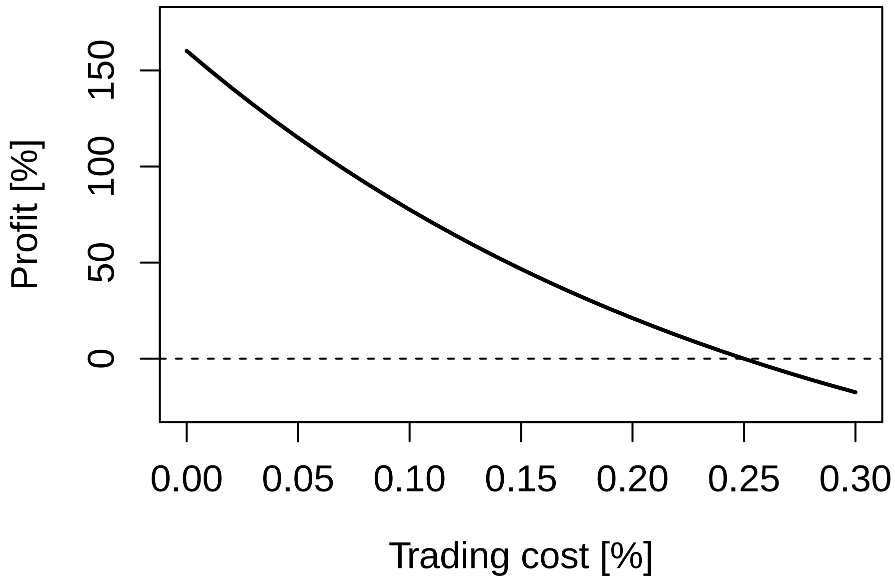

Figure 11: Final cumulative returns depending on transaction costs.

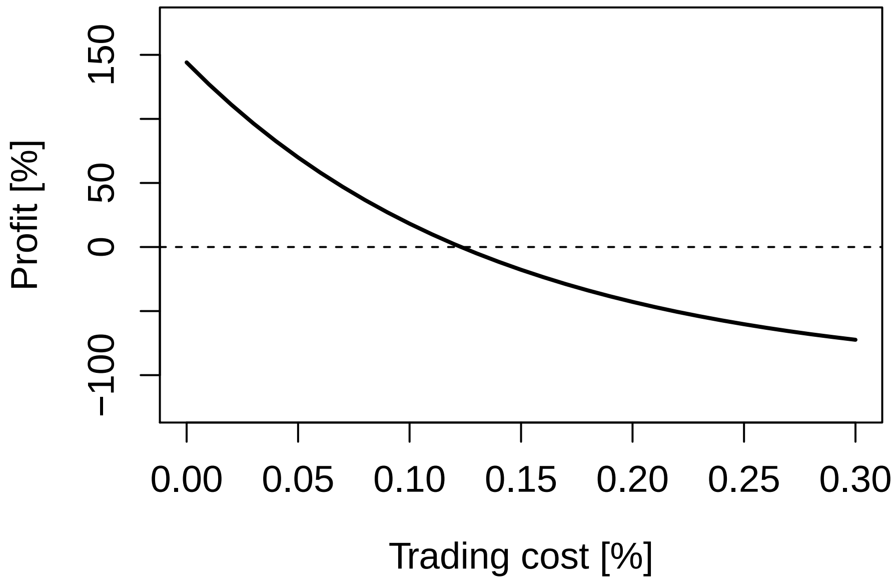

Figure 12: Final cumulative returns depending on transaction costs, for daily closing positions.

Risk Analysis

signal	ADF	ADFpval	PP	PPpval	KSPP	KSPPpval	AR
Returns	-14.87932971	0.00000000	-391.64898133	0.01000000	0.28793772	0.10000000	-0.07966048

Table 13: Stationarity tests for instantaneous returns of Combined

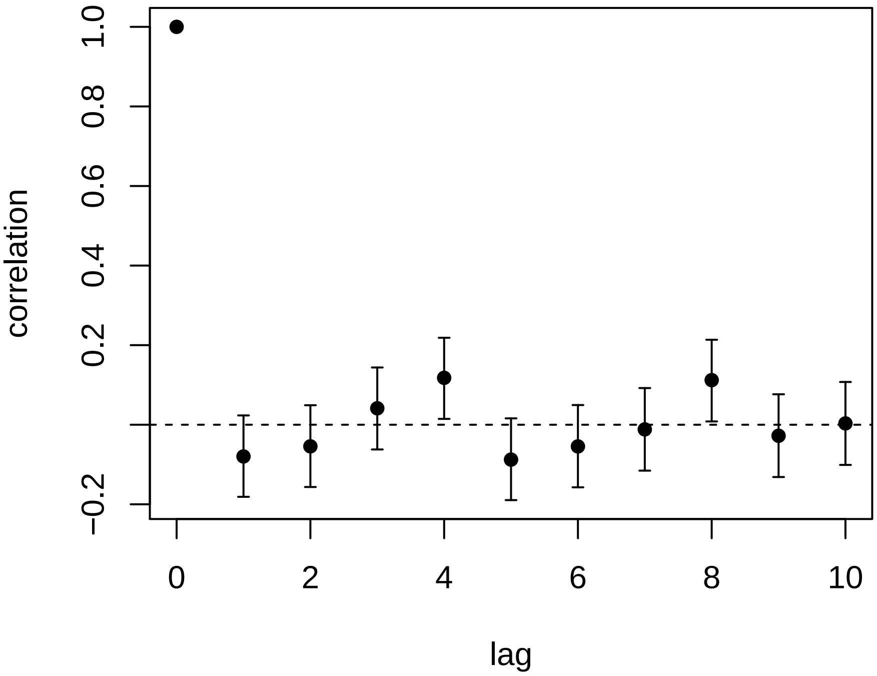

Figure 13: Autocorrelation function of Combined strategy returns

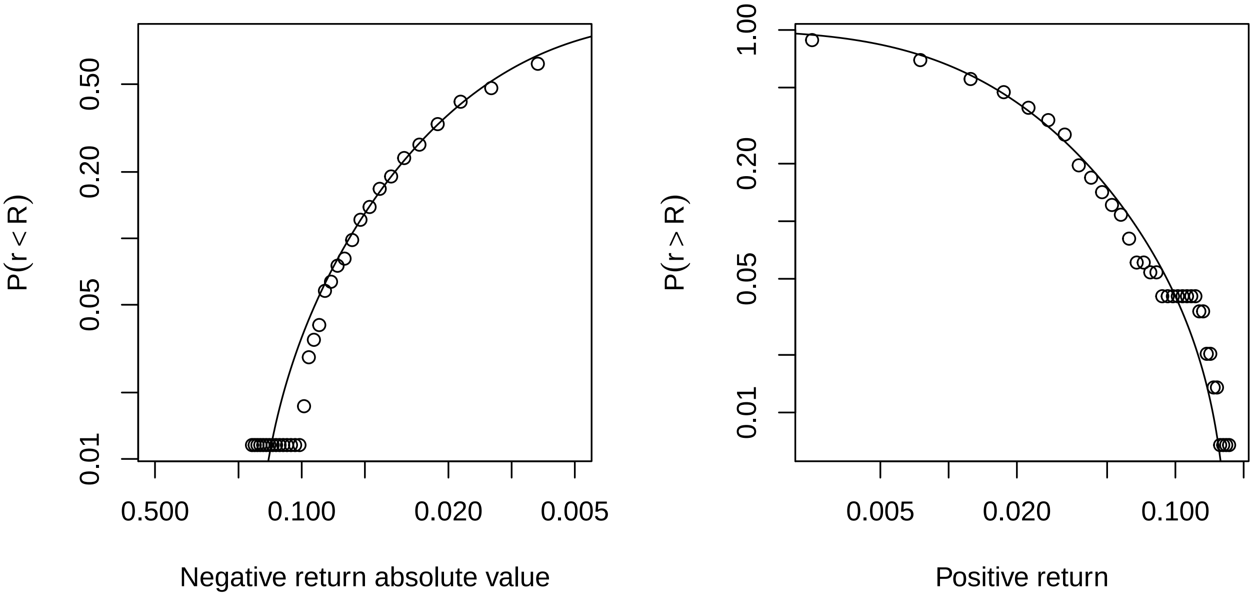

Figure 14: Distribution of Returns of Combined Strategy. The line shows the ML estimate of a lognormal fit.

- Negative Returns
  - meanlog: −4.30209144 (0.09442549)
  - sdlog: 1.24197346 (0.06676891)
  - KS Distance: 0.066764 (p-value = 0.4236)
- Positive Returns
  - meanlog: −4.11858870 (0.09595619)
  - sdlog: 1.16735747 (0.06785127)
  - KS Distance: 0.070967 (p-value = 0.4453)

Only short/long combined strategy evaluation

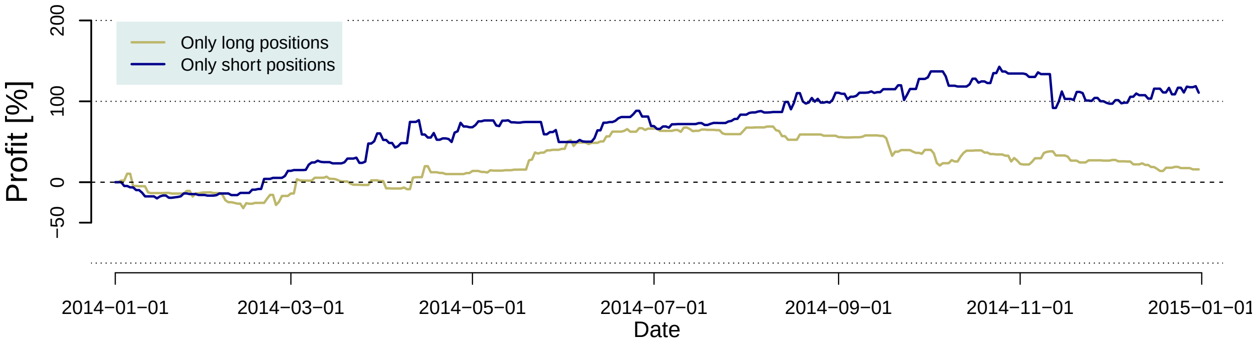

Figure 15: Time series of Combined cumulative returns when using only long and only short positions.

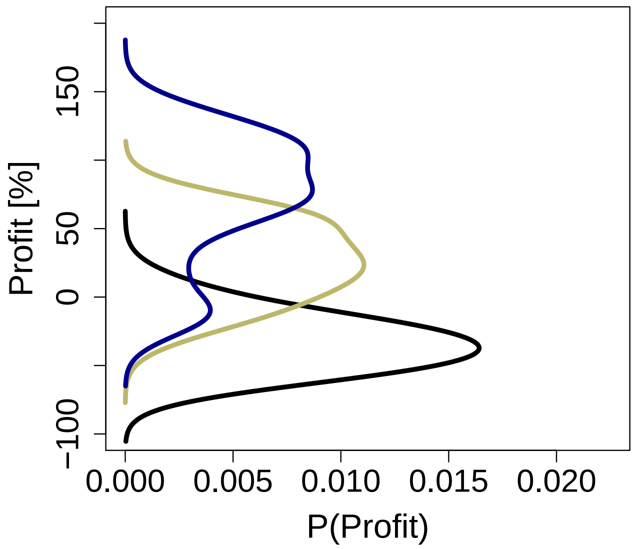

Figure 16: Kernel Density Plot of cumulative returns of data-driven strategies.
